# Exploration of the optimal number of regional lymph nodes removed for resected N0 NSCLC patients: A population-based study

**DOI:** 10.3389/fonc.2022.1011091

**Published:** 2022-09-29

**Authors:** Anjie Yao, Zixuan Liu, Hanyu Rao, Yilun Shen, Changhui Wang, Shuanshuan Xie

**Affiliations:** ^1^ Department of Respiratory Medicine, Shanghai Tenth People’s Hospital, Tongji University School of Medicine, Shanghai, China; ^2^ Tongji University School of Medicine, Shanghai, China; ^3^ Department of General Medicine, Jiuting Town Community Healthcare Cancer, Shanghai, China

**Keywords:** SEER, non-small cell lung cancer, lung cancer-specific survival, overall survival, lymph node dissection

## Abstract

**Background:**

The aim of our study was to explore the optimal number of regional lymph nodes removed (LNRs) in resected N0 non-small cell lung cancer (NSCLC) patients and identify potential risk factors.

**Methods:**

Included in this study were 55,024 N0 NSCLC patients between 2004 and 2015 based on the Surveillance, Epidemiology, and End Results database (SEER). All the patients were divided into No LNR group (57.8%), 1-3 LNRs group (8.1%) and ≥4 LNRs group (31.4%). Relevant clinical and patient parameters including overall survival (OS), lung cancer-specific survival (LCSS), gender, race, year of diagnosis, primary site, T stage, AJCC stage, laterality, histological type, lymphadenectomy, radiation, chemotherapy, age at diagnosis, insurance status, marital status, family income.

**Results:**

Kaplan-Meier analysis demonstrated LNRs had significantly better OS and LCSS than No LNRs in all the N0 NSCLC patients with different T stages (Logrank p<.001). Univariate and multivariate analysis showed that both OS and LCSS in ≥ 4 LNRs group were better than those in <1-3 LNRs group (OS: ≥4 LNRs group: HR, 0.583; 95%CI, 0.556-0.610; P<.001 vs.1-3 LNRs group: HR, 0.726; 95%CI, 0.687-0.769; P<.001; LCSS: ≥4 LNRs group: HR, 0.514; 95%CI, 0.480-0.550; P<.001 vs.1-3 LNRs group: HR, 0.647; 95%CI, 0.597-0.702; P<.001). In addition, whites, males, not upper lobe, large cell carcinoma and others, advance T stage or AJCC stage, no surgery, no LNR, no radiation, no chemotherapy, elder age at diagnosis, singled marital status and low family income had negative impact on prognosis of N0 NSCLC patients.

**Conclusions:**

Our study suggests that ≥ 4 LNRs can yield better survival outcomes compared with 1-3 LNRs in N0 NSCLC patients.

## Introduction

Lung cancer is one of the most common cancers and the leading cause of cancer-related deaths worldwide (1). About 85% of the lung cancer patients are non-small cell lung cancer (NSCLC) (2). The 5-year survival rate for NSCLC patients is approximately 15-20% (3), and therefore the opportunity for improving prognosis is pronounced and is driving advances in the diagnosis and therapy of NSCLC (4). The eighth edition of the TNM staging system is used to evaluate the NSCLC stage, which includes T (tumor size), N (nodal status), and the M (presence of metastasis), and N0 means no lymph node metastasis (5).

Historically, pulmonary resection with lymphadenectomy is the standard treatment for NSCLC patients (6). It is recommended even for the early stage of NSCLC patients—T1N0M0 patients, which may because occult lymph node metastasis and false-negative lymph nodes exist (7, 8). However, the optimal number of regional lymph nodes removed (LNRs) during surgery has remained debated all the time for the N0 NSCLC patients. The National Comprehensive Cancer Network (NCCN) indicated a minimum of 3 or more mediastinal nodal stations require examinations which can benefited patients treated by sublobar resection most (9). The American College of Surgeons Commission on Cancer (ACSCC) supported that the number of lymph nodes (LNs) examined was 10 total lymph nodes that achieve maximum benefit regardless of station for patients (10). Currently, the Union for International Cancer Control (UICC)/American Joint Committee on Cancer (AJCC) eighth edition recommends that 6 LNs is more sufficient and reliable for pathologic node staging and accurate prognostic assessment (11).

At present, the curative role of LNRs remains controversial. Few previous studies have investigated the impact of LNRs on survival for NSCLC patients, especially the N0 patients. To clarify this problem, our study aimed to explore the optimal number of regional lymph nodes removed for resected N0 NSCLC patients who were recruited from the National Cancer Institute’s Surveillance, Epidemiology, and End Results (SEER) database.

## Patients and methods

### Data source

We conducted this study to verify the relationship between the number of LNRs and the prognosis in N0 NSCLC patients. All data were based on the SEER database. As a database established in 1973, SEER collects information on cancer incidences and survival rates from the United States (US), covering 17 population-based cancer registries involving about 28% of the US population (12).

### Study population

**Figure 1:**
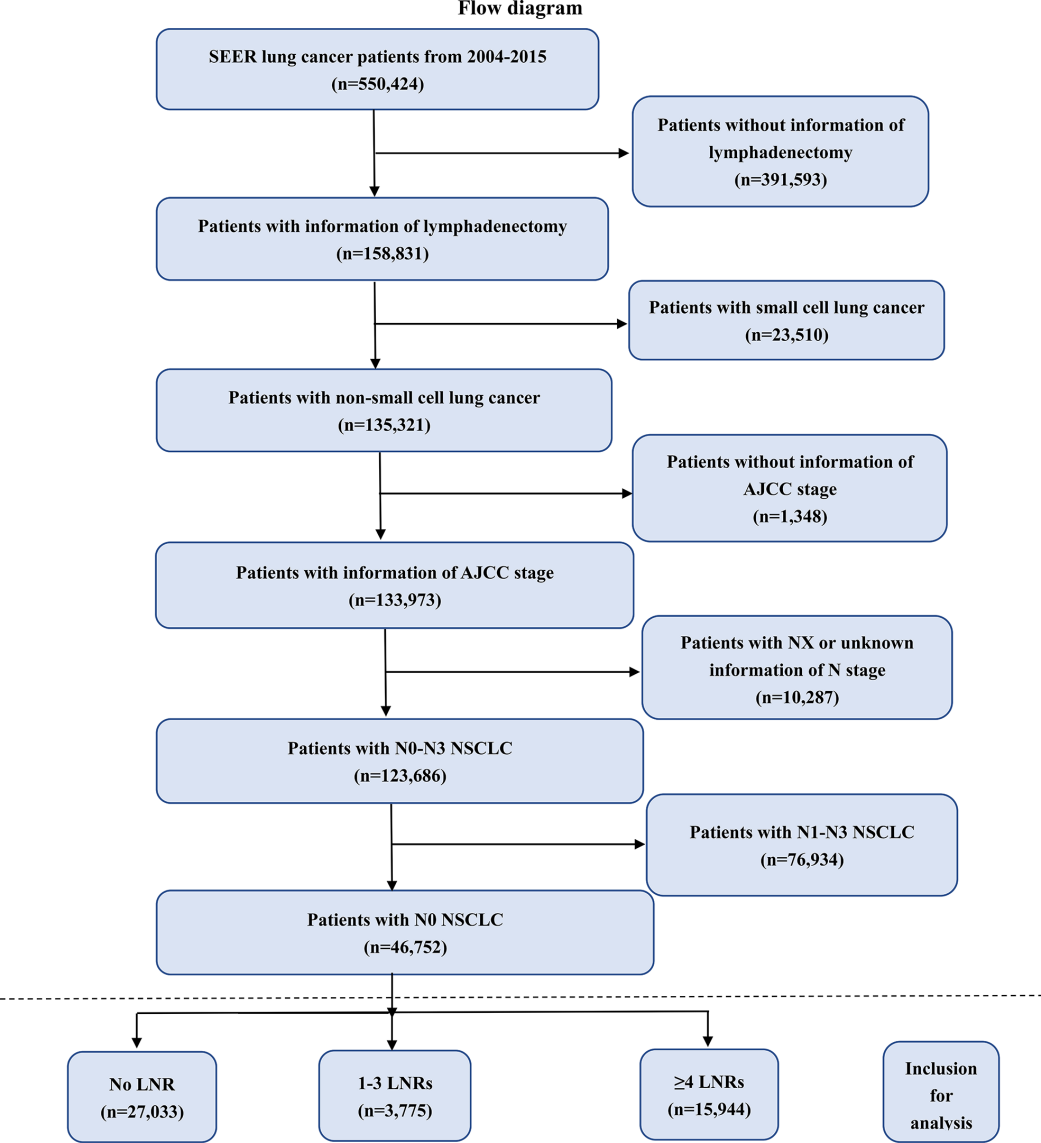
Flow chart of patient screening. NSCLC, non-small lung cancer; SEER, the Surveillance, Epidemiology, and End Results database; AJCC, American Joint Committee on Cancer; LNRs, lymph nodes removed.

A total of 550,424 lung cancer patients were identified in the SEER from 2004 to 2015 initially. We screened these patients according to our own inclusion and exclusion criteria (**Figure 1**). 46,752 patients were included, after excluding the follow patients: 391,593 patients without information of lymphadenectomy, 23,510 patients with small cell lung cancer, 1,348 patients without information of AJCC stage, 10,287 patients with unknown information of N stage and 76,934 patients with N1-N3 NSCLC tumors. We divided these included patients into three groups: No LNR (n=27,033), 1-3 LNRs (n=3,775) and ≥ 4 LNRs (n=15,944).

### Covariates

Baseline clinical characteristics of the patients, including gender, race, year of diagnosis, primary site, T stage, AJCC stage, laterality, histological type, lymphadenectomy, radiation, chemotherapy, age at diagnosis, insurance status, marital status, family income and survival months were collected and analyzed.

### Statistical analysis

T-test and chi-square test were applied to compare continuous variables and categorical variables, respectively. Survival curves were described by method of Kaplan–Meier and the survival differences between the curves were analyzed by analysis of log-rank test. In addition, Univariate and multivariate analysis was used in each group to identify other variables that had impact on survival outcomes. The forest plots were drawn to show the multivariate analysis more visually. Statistical significance was set at a two-tailed p value < 0.05. All the analysis and pictures were performed with IBM SPSS version 25.0 and GraphPad Prism version 8.0.

## Results

### Study cohort characteristics among No LNR group, 1-3 LNRs group, ≥4 LNRs group

Enrolled in this study were 46,752 patients with N0 NSCLC, including 27,033 patients with No LNRs (57.8%), 3,775 patients with 1-3 LNRs (8.1%) and 15,944 patients with ≥ 4 LNRs (34.1%) during surgery between 2004 and 2015, which indicated that the majority of all N0 NSCLC patients did not accept LNR (57.8%) during surgery and patients accepting lymphadenectomy were more likely to choose ≥4 LNRs. The baseline characteristics of these patients are shown in **Table 1**. For all the three groups, most of the patients were whites (82.0% in No LNR group; 84.9% in 1-3 LNRs group; 86.0% in ≥4 LNRs group), males (58.8% in No LNR group; 54.0% in 1-3 LNRs group; 55.6% in ≥4 LNRs group), elders (77.9% in No LNR group; 73.5% in 1-3 LNRs group; 70.9% in ≥4 LNRs group) and had no chemotherapy (63.1% in No LNR group; 74.9% in 1-3 LNRs group; 74.7% in ≥4 LNRs group). Most of the N0 NSCLC were squamous carcinoma (76.8% in No LNR group; 58.6% in 1-3 LNRs group; 58.2% in ≥4 LNRs group), T2 stage tumors (44.4% in No LNR group; 72.0% in 1-3 LNRs group; 75.9% in ≥4 LNRs group), located in upper lobe (51.4% in No LNR group; 53.8% in 1-3 LNRs group; 56.3% in ≥4 LNRs group), and had right-origin of primary (52.6% in No LNR group; 60.2% in 1-3 LNRs group; 56.9% in ≥4 LNRs group). In addition, early AJCC stage tumors were more common in ≥4 LNRs group than 1-3 LNRs group (69.2%in 1-3 LNRs group; 73.7% in ≥4 LNRs group). ≥4 LNRs group was more likely to had surgery than 1-3 LNRs group (93.1% in 1-3 LNRs group; 97.7% in ≥4 LNRs group), whereas 1-3 LNRs group was more likely to had radiation than ≥4 LNRs group (15.9%in 1-3 LNRs group; 10.4% in ≥4 LNRs group).

**Table 1:** Clinicopathological characteristics of N0 NSCLC patients.

Variable	No LNR (%) n=27033 (57.8%)	1-3 LNRs (%) n=3775 (8.1%)	≥4 LNRs (%) n=15944 (31.4%)
**Race** White Black Asian and Others	22180 (82.0%) 3516 (13.0%) 1337 (4.9%)	3206 (84.9%) 348 (9.2%) 221 (5.9%)	13710 (86.0%) 1263 (7.9%) 971 (6.1%)
**Sex** Male Female	15899 (58.8%) 11134 (41.2%)	2039 (54.0%) 1736 (46.0%)	8861 (55.6%) 7083 (44.4%)
**Year of diagnosis** 2004-2007 2008-2011 2012-2015	8106 (30.0%) 9235 (34.2%) 9692 (35.9%)	1435 (38.0%) 1278 (33.9%) 1062 (28.1%)	4937 (31.0%) 5565 (34.9%) 5442 (34.1%)
**Tumor location** Upper lobe Middle lobe Lower lobe NOS Overlapping lesion Main bronchus	13890 (51.4%) 1001 (3.7%) 8018 (29.7%) 2219 (8.2%) 278 (1.0%) 1623 (6.0%)	2031 (53.8%) 232 (6.1%) 1346 (35.7%) 78 (2.1%) 58 (1.5%) 30 (0.8%)	8969 (56.3%) 644 (4.0%) 5623 (35.3%) 235 (1.5%) 312 (2.0%) 161 (1.0%)
**Laterality** Left-origin of primary Right-origin of primary Bilateral, single primary Unknown	12250 (45.3%) 14214 (52.6%) 295 (1.1%) 274 (1.0%)	1499 (39.7%) 2272 (60.2%) 2 (0.1%) 2 (0.1%)	6856 (43.0%) 9073 (56.9%) 4 (0.0%) 11 (0.1%)
**Histology** Squamous carcinoma Adenocarcinoma Large cell carcinoma and others	20760 (76.8%) 4402 (16.3%) 1871 (6.9%)	2212 (58.6%) 1343 (35.6%) 220 (5.8%)	9279 (58.2%) 5743 (36.0%) 922 (5.8%)
**T** T1 T2 T3 T4	1349 (5.0%) 12006 (44.4%) 2679 (9.9%) 10999 (40.7%)	100 (2.6%) 2719 (72.0%) 352 (9.3%) 604 (19.0%)	256 (1.6%) 12095 (75.9%) 1501 (9.4%) 2092 (13.1%)
**Stage** I II III IV	8801 (32.6%) 1866 (6.9%) 6006 (22.2%) 10360 (38.3%)	2613 (69.2%) 317 (8.4%) 492 (13.0%) 353 (9.4%)	11752 (73.7%) 1411 (8.8%) 1826 (11.5%) 955 (6.0%)
**Surgery** No Yes Unknown	24068 (89.0%) 2748 (10.2%) 217 (0.8%)	355 (6.8%) 3514 (93.1%) 6 (0.2%)	355 (2.2%) 15583 (97.7%) 6 (0.0%)
**Radiation** No Yes Unknown	524 (1.9%) 12962 (47.9%) 13547 (50.1%)	13 (0.3%) 599 (15.9%) 3163 (83.8%)	46 (0.3%) 1652 (10.4%) 14246 (89.4%)
**Chemotherapy** No/Unknown Yes	17045 (63.1%) 9988 (36.9%)	2826 (74.9%) 949 (25.1%)	11905 (74.7%) 4039 (25.3%)
**Age at diagnosis** <65 ≥65	5974 (22.1%) 21059 (77.9%)	999 (26.5%) 2776 (73.5%)	4636 (29.1%) 11308 (70.9%)
**Insurance status** Medicaid Insured or no specifics Uninsured Unknown	2965 (11.0%) 17246 (63.8%) 457 (1.7%) 6365 (23.5%)	278 (7.4%) 2361 (62.5%) 29 (0.8%) 1107 (29.3%)	1140 (7.2%) 10854 (68.1%) 169 (1.1%) 3781 (23.7%)
**Marital status** Married/domestic partner Single/windowed/divorced Unknown	12784 (47.3%) 13270 (49.1%) 979 (3.6%)	2056 (54.5%) 1609 (42.6%) 110 (2.9%)	9398 (58.9%) 5978 (37.5%) 568 (3.6%)
**Family income** ≤5000 5000-7000 7000-9000 >9000	3863 (14.3%) 13471 (49.8%) 7044 (26.1%) 2655 (9.8%)	467 (12.4%) 1726 (45.7%) 1047 (27.7%) 535 (14.2%)	1614 (10.1%) 7287 (45.7%) 4628 (29.0%) 2415 (15.1%)

NSCLC, non-small cell lung cancer; LNRs, lymph nodes removed.

### Comparison of survival curves among No LNR group, 1-3 LNRs group, ≥4 LNRs group

**Figure 2:**
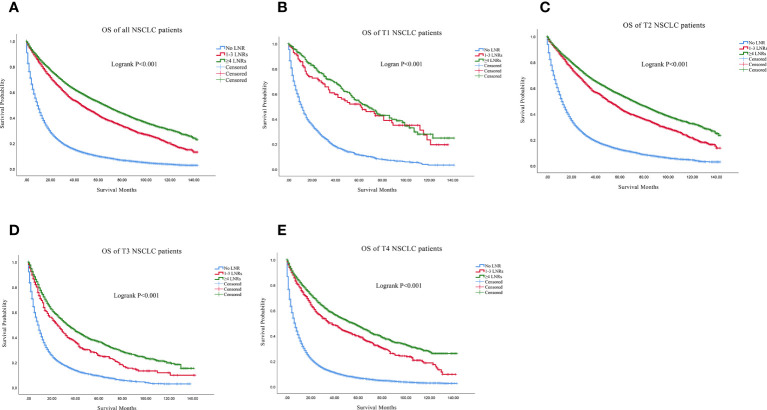
Comparison of OS in no LNR group, 1-3 LNR group and ≥4 LNRs group for N0 NSCLC patients. **(A)** Comparison in all the N0 NSCLC patients; **(B)** Comparison in T1 N0 NSCLC patients; **(C)** Comparison in T2 N0 NSCLC patients; **(D)** Comparison in T3 N0 NSCLC patients; **(E)** Comparison in T4 N0 NSCLC patients. NSCLC, non-small cell lung cancer; OS, overall survival; LNRs, lymph nodes removed.

**Figure 3:**
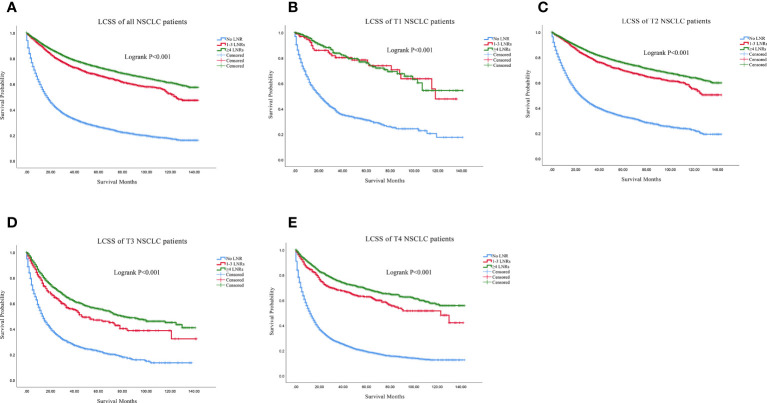
Comparison of LCSS in no LNR group, 1-3 LNR group and ≥4 LNRs group for N0 NSCLC patients. **(A)** Comparison in all the N0 NSCLC patients; **(B)** Comparison in T1 N0 NSCLC patients; **(C)** Comparison in T2 N0 NSCLC patients; **(D)** Comparison in T3 N0 NSCLC patients; **(E)** Comparison in T4 N0 NSCLC patients. NSCLC, non-small cell lung cancer; LCSS, lung cancer specific survival; LNRs, lymph nodes removed.

Firstly, we found that ≥4 LNRs group had the best 3, 5- year of OS and LCSS among the three LNR group not only in the all stage groups but only in the T1-T4 subgroups (**Table 2**). For all the N0 NSCLC patients, the Kaplan–Meier analysis demonstrated that ≥4 LNRs group had the significantly optimal OS and LCSS among the three LNR groups (**Figures 2A**, **3A**; Logrank P<.001). Furthermore, to explore the impact of LNRs count on survival of N0 NSCLC patients by different T stage, Kaplan–Meier analysis were used in T1-T4 subgroups. In T1 subgroups, the OS and LCSS curves of LNRs group were significantly better than No LNR group, whereas the survival curves of 1-3 LNRs group and ≥4 LNRs group intersected at the later survival month point (**Figures 2B**, **3B**; Logrank P<.001), which indicated that more survival analysis was needed to compare survival outcomes among the T1 subgroups. However, in T2-T4 subgroups, the OS and LCSS were significantly best in the ≥4 LNRs among the three LNR groups (**Figures 2C-E**, **3C-E**; Logrank P<.001).

**Table 2:** The 3, 5-year of OS and LCSS of N0 NSCLC patients.

	OS		LCSS	
	3-year of OS	5-year of OS	3-year of LCSS	5-year of LCSS
**All**				
No LNR	16.7%	9.8%	34.0%	26.5%
1-3 LNRs	56.0%	42.0%	74.2%	66.6%
≥4 LNRs	64.6%	52.3%	79.8%	73.0%
**T1**				
No LNR	20.5%	11.3%	37.1%	31.0%
1-3 LNRs	60.8%	51.8%	80.2%	78.3%
≥4 LNRs	71.0%	53.6%	83.7%	75.9%
**T2**				
No LNR	20.8%	12.5%	41.2%	33.1%
1-3 LNRs	59.4%	44.3%	77.7%	69.6%
≥4 LNRs	67.6%	55.0%	82.5%	75.7%
**T3**				
No LNR	15.2%	9.2%	29.3%	22.6%
1-3 LNRs	38.6%	25.5%	55.7%	46.9%
≥4 LNRs	47.8%	36.3%	63.2%	55.6%
**T4**				
No LNR	12.1%	6.8%	26.8%	19.2%
1-3 LNRs	50.1%	39.7%	68.0%	62.6%
≥4 LNRs	58.9%	47.7%	75.2%	68.8%

NSCLC, non-small cell lung cancer; LNRs, lymph nodes removed; OS, overall survival; LCSS, lung cancer specific survival.

### COX regression analysis of all the N0 NSCLC patients

**Figure 4:**
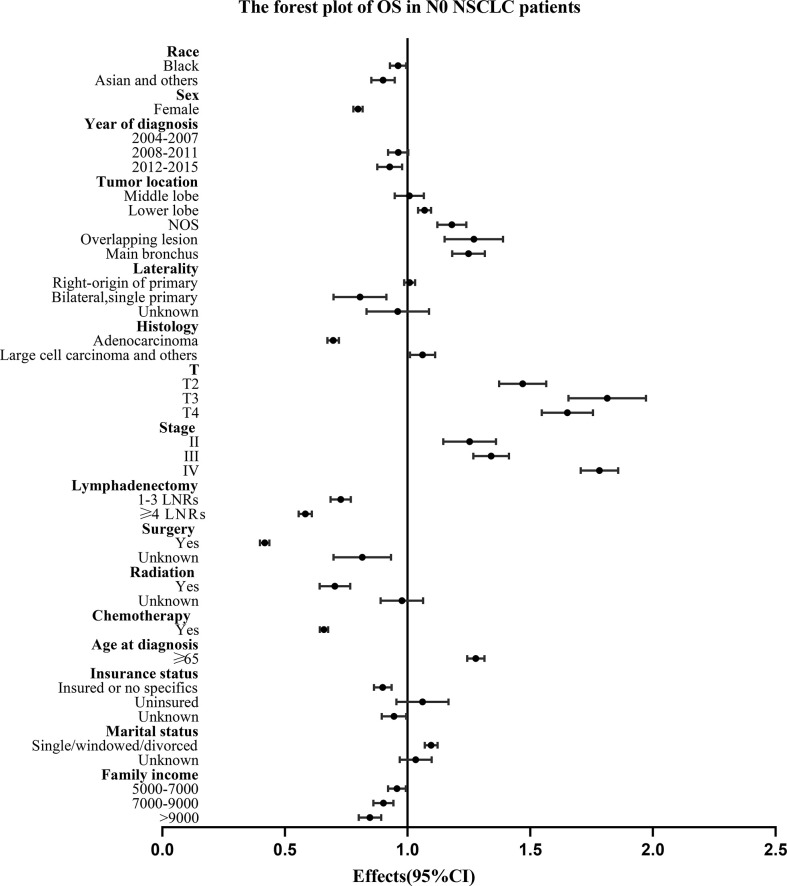
Forest plot of variables that can influence OS in N0 NSCLC patients. NSCLC, non-small cell lung cancer; CI, confidence interval; OS, overall survival; LNRs, lymph nodes removed.

**Figure 5:**
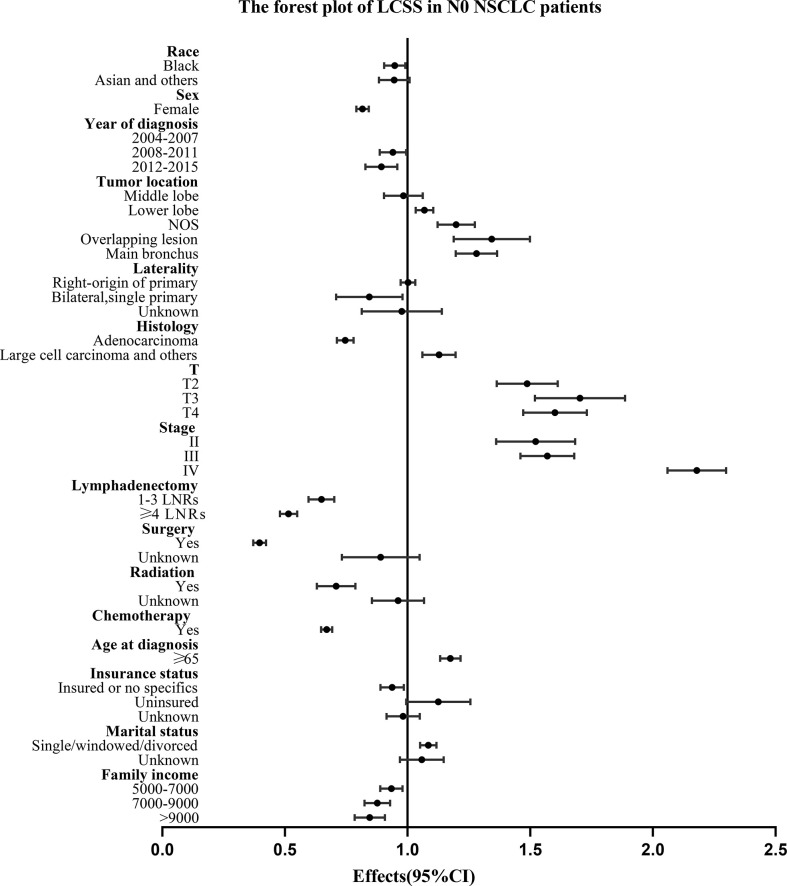
Forest plot of variables that can influence LCSS in N0 NSCLC patients. NSCLC, non-small cell lung cancer; CI, confidence interval; LCSS, lung cancer specific survival; LNRs, lymph nodes removed.

For all the N0 NSCLC patients, the Univariate and multivariate analysis demonstrated that LNR group had survival benefits than No LNR group and OS (≥4 LNRs group: HR, 0.583; 95%CI, 0.556-0.610; P<.001 vs. 1-3 LNRs group: HR, 0.726; 95%CI: 0.687-0.769; P<.001; **Table 3**) and LCSS (≥4 LNRs group: HR, 0.514; 95%CI, 0.480-0.550; P<.001 vs. 1-3 LNRs group: HR, 0.647; 95%CI, 0.597-0.702; P<.001; **Table 4**) of ≥4 LNRs group were significantly better than those of 1-3 LNRs group. In addition, the following factors had negative impact on both OS and LCSS: whites, males, not upper lobe, large cell carcinoma and others, advance T stage or AJCC stage, no surgery, no LNR, no radiation, no chemotherapy, elder age at diagnosis, singled marital status, low family income (p<.05; **Tables 3**, **4**). The forest plots showed the above survival outcomes more visually (**Figures 4**, **5**).

**Table 3:** Univariate and multivariate analyses of OS in N0 NSCLC patients.

Variables	Univariate analysis	Multivariate analysis	
	P	HR (95%CI)	P
**Race** White Black Asian and Others	**<0.001**	Reference 0.961 (0.928-0.995) 0.899 (0.853-0.948)	**<0.001** 0.025 <0.001
**Sex** Male Female	**<0.001**	Reference 0.798 (0.779-0.817)	**<0.001** <0.001
**Year of diagnosis** 2004-2007 2008-2011 2012-2015	**<0.001**	Reference 0.961 (0.921-1.003) 0.926 (0.877-0.978)	0.021 0.067 0.006
**Tumor location** Upper lobe Middle lobe Lower lobe NOS Overlapping lesion Main bronchus	**<0.001**	Reference 1.006 (0.948-1.067) 1.069 (1.043-1.096) 1.179 (1.122-1.240) 1.267 (1.153-1.391) 1.247 (1.183-1.316)	**<0.001** 0.852 <0.001 <0.001 <0.001 <0.001
**Laterality** Left-origin of primary Right-origin of primary Bilateral, single primary Unknown	**<0.001**	Reference 1.008 (0.986-1.031) 0.801 (0.701-0.916) 0.954 (0.836-1.090)	**0.008** 0.470 0.001 0.491
**Histology** Squamous carcinoma Adenocarcinoma Large cell carcinoma and others	**<0.001**	Reference 0.696 (0.673-0.720) 1.060 (1.010-1.113)	**<0.001** <0.001 0.018
**T** T1 T2 T3 T4	**<0.001**	Reference 1.467 (1.374-1.566) 1.809 (1.658-1.974) 1.649 (1.548-1.757)	**<0.001** <0.001 <0.001 <0.001
**Stage** I II III IV	**<0.001**	Reference 1.250 (1.147-1.362) 1.339 (1.269-1.414) 1.781 (1.706-1.859)	**<0.001** <0.001 <0.001 <0.001
**Lymphadenectomy** No LNR 1-3 LNRs ≥4 LNRs	**<0.001**	Reference 0.726 (0.687-0.769) 0.583 (0.556-0.610)	**<0.001** <0.001 <0.001
**Surgery** No Yes Unknown	**<0.001**	Reference 0.417 (0.398-0.437) 0.810 (0.701-0.935)	**<0.001** <0.001 0.004
**Radiation** No Yes Unknown	**<0.001**	Reference 0.702 (0.642-0.767) 0.974 (0.892-1.065)	**<0.001** <0.001 0.567
**Chemotherapy** No/Unknown Yes	**0.001**	Reference 0.659 (0.642-0.676)	**<0.001** <0.001
**Age at diagnosis** <65 ≥65	**<0.001**	Reference 1.278 (1.243-1.314)	**<0.001** <0.001
**Insurance status** Medicaid Insured or no specifics Uninsured Unknown	**<0.001**	Reference 0.898 (0.863-0.935) 1.057 (0.956-1.169) 0.944 (0.895-0.995)	**<0.001** <0.001 0.279 0.032
**Marital status** Married/domestic partner Single/windowed/divorced Unknown	**<0.001**	Reference 1.096 (1.070-1.122) 1.032 (0.969-1.099)	**<0.001** <0.001 0.323
**High school coast** ≤1000 1000-2000 2000-3000 >3000	**<0.001**	Reference 0.983 (0.953-1.015) 1.005 (0.965-1.047) 1.022 (0.948-1.102)	0.347 0.304 0.798 0.569
**Family income** ≤5000 5000-7000 7000-9000 >9000	**<0.001**	Reference 0.956 (0.921-0.993) 0.901 (0.861-0.943) 0.846 (0.801-0.893)	**<0.001** 0.022 <0.001 <0.001

NSCLC, non-small cell lung cancer; CI, confidence interval; OS, overall survival; LNRs, lymph nodes removed.

The bold indicate p values <0.05 are statistically significant.

**Table 4:** Univariate and multivariate analyses of LCSS in N0 NSCLC patients.

Variables	Univariate analysis	Multivariate analysis	
		P	HR (95%CI)	P
**Race** White Black Asian and Others	**<0.001**	Reference 0.947 (0.905-0.991) 0.944 (0.884-1.009)	**0.020** 0.019 0.091
**Sex** Male Female	**<0.001**	Reference 0.816 (0.791-0.842)	**<0.001** <0.001
**Year of diagnosis** 2004-2007 2008-2011 2012-2015	**<0.001**	Reference 0.939 (0.887-0.995) 0.892 (0.829-0.959)	**0.008** 0.032 0.002
**Tumor location** Upper lobe Middle lobe Lower lobe NOS Overlapping lesion Main bronchus	**<0.001**	Reference 0.981 (0.905-1.063) 1.068 (1.033-1.105) 1.196 (1.123-1.275) 1.337 (1.191-1.502) 1.279 (1.197-1.366)	**<0.001** 0.644 <0.001 <0.001 <0.001 <0.001
**Laterality** Left-origin of primary Right-origin of primary Bilateral, single primary Unknown	**<0.001**	Reference 1.001 (0.972-1.032) 0.837 (0.712-0.983) 0.967 (0.818-1.144)	0.185 0.943 0.030 0.698
**Histology** Squamous carcinoma Adenocarcinoma Large cell carcinoma and others	**<0.001**	Reference 0.745 (0.712-0.780) 1.127 (1.061-1.196)	**<0.001** <0.001 <0.001
**T** T1 T2 T3 T4	**<0.001**	Reference 1.484 (1.365-1.614) 1.696 (1.523-1.890) 1.598 (1.473-1.732)	**<0.001** <0.001 <0.001 <0.001
**Stage** I II III IV	**<0.001**	Reference 1.517 (1.364-1.686) 1.567 (1.462-1.681) 2.177 (2.061-2.300)	**<0.001** <0.001 <0.001 <0.001
**Lymphadenectomy** No LNR 1-3 LNRs ≥4 LNRs	**<0.001**	Reference 0.647 (0.597-0.702) 0.514 (0.480-0.550)	**<0.001** <0.001 <0.001
**Surgery** No Yes Unknown	**<0.001**	Reference 0.396 (0.371-0.423) 0.881 (0.737-1.053)	**<0.001** <0.001 0.163
**Radiation** No Yes Unknown	**<0.001**	Reference 0.706 (0.632-0.789) 0.957 (0.857-1.069)	**<0.001** <0.001 0.440
**Chemotherapy** No/Unknown Yes	**0.001**	Reference 0.670 (0.648-0.693)	**<0.001** <0.001
**Age at diagnosis** <65 ≥65	**<0.001**	Reference 1.174 (1.133-1.216)	**<0.001** <0.001
**Insurance status** Medicaid Insured or no specifics Uninsured Unknown	**<0.001**	Reference 0.936 (0.890-0.985) 1.120 (0.997-1.259) 0.981 (0.915-1.050)	**0.001** 0.011 0.056 0.575
**Marital status** Married/domestic partner Single/windowed/divorced Unknown	**<0.001**	Reference 1.084 (1.051-1.118) 1.055 (0.970-1.149)	**<0.001** <0.001 0.213
**High school coast** ≤1000 1000-2000 2000-3000 >3000	**<0.001**	Reference 1.008 (0.965-1.053) 1.042 (0.986-1.102) 1.032 (0.938-1.137)	0.375 0.719 0.140 0.518
**Family income** ≤5000 5000-7000 7000-9000 >9000	**<0.001**	Reference 0.933 (0.889-0.980) 0.875 (0.825-0.929) 0.844 (0.785-0.908)	**<0.001** 0.005 <0.001 <0.001

NSCLC, non-small cell lung cancer; CI, confidence interval; LCSS, lung cancer specific survival; LNRs, lymph nodes removed.

The bold indicate p values <0.05 are statistically significant.

## Discussion

We used the SEER Database to investigate the relevance of LNRs with prognosis in surgical resection, and the impact of the different number of LNRs on survival outcomes in N0 NSCLC patients with different T stage tumors. Our study included three N0 NSCLC groups: No LNRs, 1-3 LNRs, ≥4 LNRs. We found that patients with advanced T stage tumors were more likely to refuse LNRs. Kaplan–Meier survival analysis showed that LNRs had significantly better survival outcomes than No LNR in all N0 patients with different T stage tumors, and the beneficial impact of ≥4 LNRs on survival was evident especially in stage T2 to T4 patients. Cox-regression analysis showed that ≥4 LNRs had significantly better OS and LCSS of stage T1 to T4 patients, which suggesting that more extensive LNR may be related to better prognosis of N0 NSCLC patients no matter what T stage was. There were some risk factors to survivals of those patients: whites, males, early year of diagnosis, not upper lobe, large cell carcinoma and others, advanced T stage, no LNR, no surgery, no radiation, no chemotherapy, elderly age at diagnosis, uninsured, singled status, and low family income.

Generally, surgical resection with lymphadenectomy was the standard treatment for the early and locally advanced NSCLC patients (13). Even in the N0 NSCLC, LNR was still necessary due to the pathologic assessment and survival benefits (7), which is consistently with our view that LNRs could contribute to longer survival time than no LNR. We also found that a larger number of LNRs may be related to better survival outcomes in N0 NSCLC patients. A randomized Z0030 trial enrolling 1,111 NSCLC patients demonstrated that LNRs did not increased morbidity or mortality in early-stage patients including N0 patients (14). But another observational study divided 2,047 NSCLC patients into 8 sequentially more thorough lymphadenectomy groups, and found that 5-year survival of N0 patients improved sequentially from the least extended group (HR: 0.63,95% CI: 0.59-0.66) to the most extended group (HR: 0.71, 95% CI: 0.60-0.79) (15), which is also consistently with our view.

The number of LNRs could be associated with the accuracy of nodal staging and the survival prognosis of the NSCLC patients (16, 17). However, the optimal number of LNRs in the NSCLC patients has been debated for a long time (18). In our study, we found ≥4 LNRs had significantly better survival outcomes than 1-3 LNRs in N0 NSCLC patients with T1 to T4 tumors, so we recommended ≥4 LNRs in any stage of N0 NSCLC patients including stage I to IV. A population study analyzed stage I to IIIA resected NSCLC data from a Chinese multi-institutional registry (n=5,706) and the US SEER database (n=38,806), and demonstrated that a larger number of LNRs was positively related to better OS in N0 patients (SEER: HR, 0.986; 95% CI, 0.983 to 0.989; *P* <.001; China: HR, 0.981; 95% CI, 0.972 to 0.989; *P* <.001), and ≥16 LNRs could reduce all-cause mortality of N0 NSCLC patients significantly (derivation cohorts: SEER 2001 to 2008 HR, 0.830; China HR, 0.738; SEER 2009 cohort: HR, 0.837) (19). Another study recruited 1,205 resected stage I-II NSCLC patients from 6 Chinese institutions, and demonstrated that 6 LNRs was the optimal number of nodal stations removed and ≥6 LNRs could reduce all-cause mortality significantly (20). Notably, we demonstrated that ≥4 LNRs also had significantly survival benefits to stage IA NSCLC patients with T1N0M0 tumors when compared to 1-3 LNRs. Consistently with our view, a recent research based on 3,269 patients with stage IA NSCLC tumors ≤2 cm indicated that 1-3 LNRs had significantly worse OS (HR, 1.319; 95% CI, 1.065-1.634; *P* = .011) or LCSS (HR, 1.396; 95% CI, 1.034-1.885; *P* = .029) than ≥4 LNRs in patients after sublobar resection (21), and another study enrolling 9,603 T1a-1b N0 M0 NSCLC patients also concluded that 4 was the optimal cutoff value for LNRs count (p <.0001) and ≥4 LNRs was significantly related to better OS (HR: 0.741; 95% CI: 0.679–0.810; p <.001) and LCSS (HR: 0.710; 95% CI: 0.629–0.802; p <.001) rather than <4 LNRs (22). Similarly, another retrospective research evaluated 1,420 stage IA-IIB NSCLC, N0 patients after lobectomy and reported that ≥3 LNRs (HR, 0.68; P = 0.013) was significantly associated with better survival prognosis compared to <3 LNRs (23). However, another study based on 65,438 stage I NSCLC patients’ data from the National Cancer Database illustrated that 8, 9, 10, 11 LNRs was optimal for prognostic stratification in T1a (HR = 0.718, P = 1.56E–04), T1b (HR = 0.880, p = 7.17E–04), T1c (HR = 0.869, P= 9.04E–04) and T2a (HR = 0.859, P = 6.11E–05) tumors which indicated that increasing number of LNRs was associated with better survival outcomes (24). What’s more, besides tumor size and tumor stage, the appropriated number of LNRs in resected N0 NSCLC patients was affected by many other factors, such as organ metastasis and operation ways (25–27). It was necessary to have more further prospective studies to explore the optimal number of LNRs.

Why more extensive excision of LNRs was beneficial even to the N0 NSCLC patients with stage I-IV tumors? There are some plausible potential reasons to explain these findings. First, routine sentinel nodal examinations may not include all the relevant pathways and routine pathology may not correctly differentiate nodes affected by the tumor (28). Second, more extensive LNRs may reflect better surgical skills of the doctors and the appropriateness of pathological, surgical and specialized care offered by the medical team, and therefore would affect the outcomes of treatments (29). Finally, the tumor microenvironment may have changed before the tumor appeared, in which tumor genetic material may cause tumor recurrence and nodal metastasis (30).

Compared with other studies, our study comprised a relatively large cohort of N0 NSCLC patients in multiple centers with real-world datasets with robust statistics. But our study also has several limitations. First, this research was retrospective in nature which may cause some data missing and study bias. Also, there was a lack of original data of our own study. In addition, the SEER database does not provide some details affecting lymphadenectomy, such as the specific type of surgery, ways of lymphadenectomy, distant organ metastasis, disease-free survival, local progression-free survival and underlying diseases. However, with 15 variables and total 46,752 patients, our study still represents a scientific analysis of LNRs for N0 NSCLC patients with T1-T4 tumors. Therefore, our findings can provide constructive suggestions about preoperative examination, clinical operation and postoperative nursing for N0 NSCLC patients in the future.

## Conclusion

The results of the present study demonstrated that the number of LNRs affected the prognosis of N0 NSCLC patients. The more count of LNRs was correlated with better OS and LCSS. We recommended ≥ 4 LNRs in all the N0 NSCLC patients with stage T1-T4 tumors because it contributes better prognosis compared to 1-3 LNRs.

## Data availability statement

The datasets presented in this study can be found in online repositories. The names of the repository/repositories and accession number(s) can be found in the article.

## Author contributions

Conception and design: AY, ZL, HR, YS, SX, and CW. Acquisition, statistical analysis, or interpretation of the data: all authors. Drafting of the manuscript: AY, ZL, HR, SX, and CW. All authors reviewed and approved the final version of the manuscript.

## Acknowledgments

We would like to thank all the patients who donated their statistical data.

## Conflict of interest

The authors declare that the research was conducted in the absence of any commercial or financial relationships that could be construed as a potential conflict of interest.

## Publisher’s note

All claims expressed in this article are solely those of the authors and do not necessarily represent those of their affiliated organizations, or those of the publisher, the editors and the reviewers. Any product that may be evaluated in this article, or claim that may be made by its manufacturer, is not guaranteed or endorsed by the publisher.

## Abbreviations

SEER, the Surveillance, Epidemiology, and End Results; OS, Overall Survival; LCSS, Lung cancer specific survival; NSCLC, Non-Small Cell Lung Cancer; LNRs, Regional lymph nodes removed.
